# Low FT4 levels in early pregnancy are associated with higher insulin therapy need in women with gestational diabetes mellitus

**DOI:** 10.1530/ETJ-25-0206

**Published:** 2026-01-08

**Authors:** Emna Jelloul, Manon Lomré, Pierre Kleynen, Georgiana Sitoris, Lidia Grabczan, Flora Veltri, Madhu Prasai, Serge Rozenberg, Kris G Poppe

**Affiliations:** ^1^ Department of Endocrinology, Centre Hospitalier Universitaire (CHU) Saint Pierre, Université Libre de Bruxelles (ULB), Brussels, Belgium; ^2^ Departement of Gynaecology and Obstetrics, Centre Hospitalier Universitaire (CHU) Saint Pierre, Université Libre de Bruxelles (ULB), Brussels, Belgium

**Keywords:** gestational diabetes, thyroid disorders, insulin therapy

## Abstract

**Objective:**

To compare demographical and thyroid parameters/disorders between a group of women with gestational diabetes mellitus (GDM) treated with diet and lifestyle measures (DiLS) and a group also necessitating insulin therapy (IT). Furthermore, to determine which variables were associated with IT need.

**Design/methods:**

Retrospective analysis in a single centre cohort including 1,782 women screened at median 12 (11–14) weeks of pregnancy for thyroid disorders (TSH, free T4 (FT4), and TPOAb) and for GDM with an oral glucose tolerance test at 24–28 weeks. Women with assisted and multiple pregnancies were excluded (*n* = 85). The diagnosis of GDM was based on the 2013 WHO criteria, and TAI on increased TPOAb levels (≥60 kIU/L). Multivariable regression analyses were applied to identify variables/conditions (thyroxine treatment before or after screening, thyroid parameters (TSH and FT4 levels), and thyroid disorders (subclinical hypothyroidism (SCH) and/or thyroid autoimmunity (TAI)) were associated with IT.

**Results:**

Overall, 328 of the 1,697 women were diagnosed with GDM (19.3%); 54 were treated with IT (16.5%) and 274 only with diet and lifestyle measures (83.5%). After adjustment for confounders, only FT4 levels were associated with IT need (adjusted odds ratio: 0.76; 95% CI: 0.61–0.94; *P* = 0.011).

**Conclusion:**

Lower serum free T4 levels were independently associated with a higher risk for IT need in women with GDM. More research is needed to unravel this association, but insulin resistance is probably a major contributor.

## Introduction

Gestational diabetes mellitus (GDM) is a prevalent complication during pregnancy (11% in Europe doi: 10.3389/fendo.2021.691033.) and known to be associated with higher rates of preeclampsia, macrosomia, and other complications (1). Similarly, thyroid disorders during pregnancy, including (subclinical) hypothyroidism (SCH) and thyroid autoimmunity (TAI), are common and linked to various adverse pregnancy outcomes, of which some are the same as documented in case of GDM (2).

Thyroid disorders have been associated with a higher risk of GDM via different pathways, due to increased insulin resistance (IR) in case of thyroid dysfunction, and via common inflammatory pathways in case of the presence of TAI (3, 4).

The initial treatment of GDM consists in dietary (Di) and lifestyle (LS) measures (DiLS), and when glycaemic targets as proposed by the international societies are not rapidly reached, insulin therapy (IT) is started (5).

Several factors have been associated with the need for IT in GDM, including advanced maternal age, body mass index (BMI) (obesity) and a history of GDM or familial diabetes (6).

Data on the impact of thyroid dysfunction on the IT need in women with GDM are limited to three studies (7, 8, 9). One in association with women who had hypothyroidism (7) and two others in case of hypothyroxinaemia (8, 9).

We had two main aims in this study. The first was to compare all baseline data (demographic, obstetric and thyroid) and thyroid disorders between women with GDM in the DiLS group versus the IT group, and the second to determine in logistic regression analyses which independent variables (demographic, obstetric and thyroid parameters/disorders and treatment) were associated with IT need in women with GDM as dependent outcome.

## Subjects and methods

### Study population and aims

This cohort study results from a collaboration between the departments of Endocrinology and Gynaecology and Obstetrics in CHU Saint Pierre, a public university hospital in Brussels, Belgium. The study was approved by the institutional review board ‘Comité Local d’Éthique Hospitalier, No d’agréation: O.M. 007 AK/15-11-114/4568’. The need for written consent from study participants was waived due to the retrospective analysis of routinely collected data.

In our centre, a systematic screening for GMD with an oral glucose tolerance test (OGTT) is done (when normal first trimester glycaemia). This is done since in our centre, many women have a high risk profile (obese, older age, and other background than Caucasian), with a reported prevalence of GDM of 19.6% (10). Eligibility criteria comprised all women with ongoing pregnancies who performed their laboratory screening (including the OGTT) and full obstetric follow-up in our centre from the beginning of January 2013 to the end of December 2014. We included only women who performed an OGTT.

**Figure 1:**
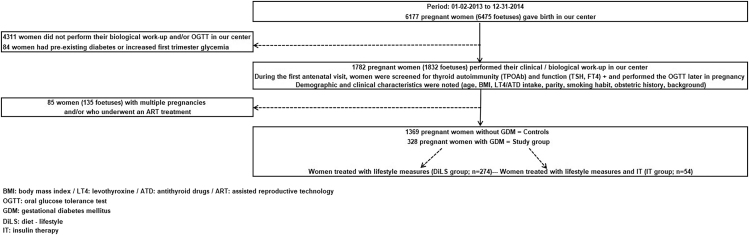
Flowchart of the study selection process.

As shown in Fig. 1, women with pre-existing diabetes, pregnant after IVF/ICSI or multiple pregnancies were excluded; finally, 328 women with GDM were stratified into two groups: group-1 (*n* = 274) ‘DiLS’ treated only with diet and lifestyle measures, and group-2 (*n* = 54) ‘IT’, who needed on top of the DiLS also IT to achieve correct glycaemic targets.

The first aim of the study was to investigate if demographic data, obstetric history, thyroid disorders/treatment and parameters (TSH, FT4 and TPOAb) were different between both study groups.

The second aim of the study was to determine variables (demographic, obstetric and thyroid) that were associated with the main study outcome, namely IT in women with GDM.

For the logistic regression analyses, from the 328 women with GDM, 57 patients were excluded (seven with suppressed serum TSH, three with isolated FT4 levels and 46 who were screened for thyroid disorders >20 weeks of gestation); the regression analyses were performed on the data of the remaining 272 women.

Associations with the depended outcome (IT need in women with GDM) were made with thyroxine treatment (for SCH) initiated before or after screening, thyroid parameters (TSH and FT4 levels), and thyroid disorders (SCH or TAI). For TAI, we applied a first trimester determined cut-off for TPO (≥60 kIU/L) (11).

Corrections were made for a history of GDM, parity, maternal age and BMI, tobacco use in pregnancy and foetal gender.

### Methods

#### Definitions

During the first antenatal consultation, demographic parameters and obstetric data were collated and routine blood tests, including serum TSH, FT4, thyroid peroxidase antibodies (TPOAbs) and fasting glucose. An OGTT was performed at 24–28 weeks of pregnancy.

Gestational age was based on ultrasound findings and expressed in full weeks and days of amenorrhoea. Smoking was stratified as yes/no (women who stopped smoking during pregnancy were also considered as smokers).

GDM was diagnosed by a 75 g OGTT performed between 24 and 28 weeks of pregnancy according to the WHO criteria: fasting glucose ≥92 mg/dL or 1 h postprandial glucose ≥180 mg/dL or 2 h ≥153 mg/dL) (12). TAI was defined as TPOAb levels ≥60 kIU/L, and SCH when TSH levels were ≥3.74 mIU/L.

A dietary consultation and lifestyle instructions were provided. We proposed glycaemic tests four times a day; in the morning on an empty stomach and 2 h after each meal. One week to 10 days later, blood glucose profiles were assessed. Long-acting insulin was started in the evening if fasting blood glucose remains ≥95 mg/dL in 20–30% of results, and rapid insulin was started if it remains >20–30% of postprandial blood glucose levels or were ≥120 mg/dL at 2 h (5).

#### Serum assays

All blood test analyses were performed by the laboratory of CHU Saint Pierre.

Serum TSH, FT4 and TPOAb levels were measured using the Chemiluminescence Centaur XP Siemens immunoanalyzer. Nonpregnant reference values were 0.3–4.0 mIU/L, 10.3–25.7 pmol/L and <60 kIU/L for TSH, FT4 and TPOAb, respectively. Total imprecision CVs were 6.9, 4.2 and 7.6% for TSH, FT4 and TPOAb, respectively.

In a Danish study, it was determined that in pregnant women (median pregnancy week 10 (range, 4–20)), for the same assay, the 95th cut-off for TPOAb was 60 kIU/mL (11).

In a previous study, we determined institutional-specific first-trimester reference ranges for serum TSH and FT4, according to the ATA-GL recommendations (13). The reference range for serum TSH (2.5–97.5th percentile) was 0.06–3.74 mIU/L, and 10.29–18.02 pmol/L for serum FT4. Plasma glucose was measured by an automated colourimetric-enzymatic method on a Hitachi/Roche-Modular P analyser; CV was 1%.

#### Statistical analysis

Data were stored in a Microsoft Excel database and statistical analyses performed using StatPlus:mac, Analyst Soft Inc. – statistical analysis program for macOS; version v8 (https://www.analystsoft.com/en/). Descriptive statistics are presented as the mean ± standard deviation (SD) for normally distributed and median (interquartile range (IQR)) for skewed variables. Comparison between groups was performed by Chi^2^ or Fisher’s exact tests (according to the number of events) for categorical data and by Student’s *T*-test or Mann–Whitney U test for continuous data (according to the distribution). In the section on the aims, we explained how the logistic regression analysis was performed.

*P* values were considered significant whenever *P* < 0.005.

## Results

Table 1 shows baseline demographic and obstetric parameters in all women with GDM and according to the treatment type.

**Table 1:** Demographic and obstetric parameters in women with GDM and according to the treatment type. Continuous data are expressed as the mean ± SD or median (IQ 1–3), according to the distribution and categorical data as *n* (%). Statistically significant values are presented in bold.

Demographic and obstetric data	All GDM women	DiLS	IT	*P*-level
*n*	328 (100%)	274 (83.5%)	54 (16.5%)	
Maternal age (years)	32 (28–36)	32 (28–36)	32 (29–35)	0.856
Maternal (age ≥30 years)	211 (64.3%)	177 (64.6%)	34 (63%)	0.819
BMI pre-pregnancy (kg/m^2^)	27 (23–30)	26 (23–30)	28 (25–33)	**0.007**
Obesity (BMI ≥ 30 kg/m^2^)	90 (27.4%)	68 (24.8%)	22 (40.7%)	**0.017**
Background (other than Caucasian)	271 (82.6%)	224 (81.8%)	47 (87%)	0.349
Parity	1 (0–2)	1 (0–2)	0.5 (0–2)	**0.020**
Multiparity (≥2)	123 (37.5%)	109 (39.8%)	14 (25.9%)	0.055
History of ≥2 first trimester MC	27 (8.2%)	25 (9.1%)	2 (3.7%)	0.185
Previous episode of GDM	25 (7.9%)*	17 (6.5%)	8 (14.8%)	**0.040**
Smoking during pregnancy	48 (14.6%)	42 (15.3%)	6 (11.1%)	0.423
Fetal gender (female)	167 (50.9%)	139 (50.7%)	28 (51.9%)	0.880

DiLS, diet – lifestyle; IT, insulin therapy; GDM, gestational diabetes mellitus; BMI, body mass index; MC, miscarriage.

* *n* = 315.

The median (IQR) age for all women was 32 (28–36) years, and comparable between both study groups (*P* = 0.856). The median BMI was 27 (23–30) kg/m^2^ for all women and higher in GDM-IT vs GDM-DiLS women (28 (25–33) vs 26 (23–30) kg/m^2^; *P* = 0.007). Similarly, a higher prevalence of obesity (BMI ≥ 30 kg/m^2^) was noted (40.7 vs 24.8%; *P* = 0.017).

Women in the GDM-IT group had a lower prevalence of high parity rate (≥2) vs those in the GDM-DiLS group (25.9 vs 39.8%; *P* = 0.020).

No difference was noted between groups concerning the prevalence of other than the Caucasian background, smoking during pregnancy, history of miscarriage or fetal gender.

Table 2 shows thyroid parameters in all women with GDM and according to the treatment. Gestational age at blood sampling was 13 (11–16) weeks, and comparable between both study groups (*P* = 0.242). The only parameter that tended to be different between both groups was serum FT4 (12.9 (11.6–14.2) in the GDM-IT vs 14.2 (12.9–15.1) in the GDM-DiLS group; *P* = 0.066). All other thyroid parameters and the prevalence of disorders were comparable between both study groups.

**Table 2:** Thyroid parameters in women with gestational diabetes mellitus (GDM), according to the treatment type. Continuous data are presented as the mean ± SD or median (IQ 1–3), depending on the distribution.

Demographic & obstetric data	All GDM women	DiLS	IT	*P*-level
*n*	328 (100%)	274 (83.5%)	54 (16.5%)	
Gestational age at blood sampling (weeks)	13 (11–16)	13 (11–16)	12 (11–14)	0.242
TSH (mIU/L)	1.48 (0.93–2.19)	1.48 (0.94–2.18)	1.37 (0.88–2.27)	0.958
FT4 (pmol/L)	14.2 (12.9–14.2)	14.2 (12.9–15.1)	12.9 (11.6–14.2)	0.066
TPOAb (kIU/L)	29 (28–39)	29 (28–40)	28 (28–34)	0.337
TAI (TPO ≥ 60 kIU/L)	31 (9.5%)	28 (10.2%)	3 (5.6%)	0.284
(Sub) clinical hyperthyroidism	7 (2.1%)	7 (2.6%)	0 (0%)	0.605
(Sub) clinical hypothyroidism	25 (7.6%)	20 (7.3%)	5 (9.3%)	0.620
Known thyroid disorder before screening	9 (2.7%)	7 (2.6%)	2 (3.7%)	0.637
LT4 treatment after screening	34 (10.4%)	28 (10.2%)	6 (11.1%)	0.844

DiLS, diet – lifestyle; IT, insulin therapy; GDM, gestational diabetes mellitus; TSH, thyrotropin; FT4, free thyroxine; TPOAb, thyroid peroxidase autoantibody; TAI, thyroid autoimmunity; TD, thyroid disorder; and LT4, levothyroxine.

Table 3 shows the results of the multivariable logistic regression analyses.

**Table 3:** Multivariable logistic regression analyses, with IT need in GDM as dependent outcome.

Independent variables	aOR	95% CI	*P*-level
Thyroid condition			
Treatment before pregnancy	1.26	0.21–7.74	0.802
Treatment after screening	1.05	0.37–2.95	0.934
TAI (TPOAb ≥ 60 kIU/L)	0.54	0.11–2.57	0.435
TSH (all levels)	0.99	0.73–1.34	0.944
SCH	1.40	0.41–4.86	0.592
FT4 (all levels)	0.76	0.61–0.94	**0.011**

IT, insulin therapy; GDM, gestational diabetes mellitus; aOR, adjusted odds ratio; 95% CI, 95% confidence interval; TSH, thyrotropin; FT4, free thyroxine; TPOAb, thyroid peroxidase autoantibody; TAI, thyroid autoimmunity; BMI, body mass index; and SCH, subclinical hypothyroidism.

Only FT4 levels were associated with IT need in women with GDM; aOR: 0.76; 95% CI: 0.61–0.94; *P* = 0.011. Correction for TAI (considering a cut-off ≥60 kIU/L) did not modify the association.

Other results not shown in the tables:

If the cohort of patients who were included to perform the regression analyses (*n* = 272) was divided into three tertiles according to their BMI’s, the prevalence of IT women in T3 (BMI: 30–51 kg/m^2^; *n* = 73) was 14 vs 27% in T1 (BMI: 17–25 kg/m^2^; *n* = 107); *P* = 0.026. The prevalence between the other tertiles was comparable (data not shown). The FT4 values were comparable between T3 and T1 (12.9 (11.9–15.4) vs 14.2 (12.9–14.2) pmol/L); *P* = 0.741.

When the logistic regression analyses were done for each tertile of BMI separately, only for tertile-2 (the normal BMI group), an association was obtained between FT4 levels and the dependent outcome IT (aOR: 0.53; 95% CI: 0.31–0.90; *P* = 0.018). For the other tertiles, no significant results were observed (data not shown).

## Discussion

The major study result was the association between lower FT4 levels and the higher prevalence of women who needed IT on top of dietary and lifestyle measurements to control their GDM, according to the international standards (5).

In several studies, low FT4 levels have been associated with a higher prevalence of GDM, but IT treatment as outcome was not considered (14, 15, 16, 17, 18, 19).

In addition, beyond the scope of GDM, in a longitudinal study on the progression from prediabetes to diabetes type 2, it was shown that the risk was higher with low-normal thyroid function (HR = 0.91; 95% CI: 0.86–0.97 for FT4) (20), and in another study, subjects with the highest FT4 quartiles showed significantly lower fasting insulin and HbA1c levels (21).

On the other hand, in some studies, no association between low FT4 levels and a higher risk of GDM or IR was noted (22, 23). This discrepancy can be explained by a different study setting, other assays for FT4, other gestational ages at which FT4 was measured, heterogeneity in the definition of GDM, and others.

Several mechanisms have been proposed to explain the association between low(er) FT4 levels and a higher prevalence of GDM. In the study by Zhang *et al.*, it was noted that triglyceride levels mediated 21.3% of the association between FT4 and GDM; high triglycerides are known to affects insulin sensitivity (15). Thyroid hormone (TH) receptors are present in pancreatic β-cells and TH can stimulate β-cell proliferation and insulin secretion, important to maintain normal glucose homoeostasis during pregnancy (24). In peripheral tissues, T3 increases glucose transporter type 4 (GLUT4) expression and mitochondrial oxidative capacity, thereby facilitating glucose uptake and utilization. A reduction in circulating FT4 may lead to insufficient T3, contribute to β-cell dysfunction and peripheral IR (25, 26).

TH influences glucose metabolism and may contribute to the development of GDM. With increased TH availability, glucose uptake in skeletal muscle is enhanced, endogenous glucose production rises, and insulin half-life is reduced. Both *in vitro* and clinical studies demonstrate that either low or high TH availability can increase IR. An alternative explanation is that isolated hypothyroxinaemia/lower FT4 levels may reflect a non-thyroidal illness process, linking it to GDM through inflammatory or metabolic pathways. It was observed that low FT4 was associated with higher fasting insulin levels, but not with fasting glucose, suggesting that increased IR in participants with low FT4 was compensated for by elevated insulin secretion. However, lower FT4 was associated with higher post-load glucose concentrations during the OGTT, indicating that compensatory mechanisms were insufficient to fully counteract the IR. Interestingly, the association between FT4 and non-fasting glucose during the OGTT varied by maternal BMI, with attenuation at higher BMI, suggesting that the influence of TH on IR may be less important compared to f.i. obesity (17, 18, 19).

This is nicely shown when we divided the cohort into three tertiles according to the BMI. The impact of FT4 on IT need was only present in the tertile with normal BMI levels. In addition, in the meta-analysis, the odds of BMI in relation to IT need was much stronger compared to that of FT4 levels (data not shown).

The placenta plays an important role in the relationship between FT4 and GDM via two mechanisms. On the one hand, and excessive type 3 deiodinase, which inactivates T4 and T3, and low(er) maternal FT4 may impair placental development and function, resulting in altered nutrient transport, increased inflammation, and oxidative stress – mechanisms implicated in GDM pathogenesis (27). On the other hand, in case of an impaired placental function, lower hCG levels will stimulate less the thyroid gland, and therefore, FT4 levels will be lower and the prevalence of GDM higher (14, 16).

Previous studies have shown that euthyroid women with TAI had an increased risk of GDM (4, 28). The underlying hypotheses are that common variants in immune genes might be involved and/or that the cytokines involved in TAI (IL-6 and TNF-α) might lead to a higher degree of IR (3, 29, 30). However, in our study, women in the GDM-DiLS group tended to have a higher prevalence of TAI compared with women in the GDM-IT group. This might be an argument in favour of the impact of TH/low(er) FT4 levels as the more important underlying mechanism, compared with that of a common inflammatory pathway. Furthermore, some studies did not observe higher rates of GDM in women with TAI (31).

Limitations of our study were that the retrospective design though the data were prospectively collected, the absence of hCG levels, TgAb status, and FT3 levels. Concerning hCG, it is known that it stimulated thyroid function and that higher levels are associated with less GDM (16). TgAb positivity, which can occur isolated in about 5% of women without TPOAb positivity has also been associated with an impaired impact of hCG on thyroid function during pregnancy (32, 33). However, TgAb positivity has not been associated with lower FT4 levels (34).

Some studies associated a higher FT3-to-FT4 ratio or only FT3 levels during pregnancy with GDM, and no association with FT4 levels (35).

However, the measurement of FT3 levels during pregnancy remains debated (36).

## Conclusion

Women with lower FT4 levels, a lower parity rate and a history of GDM had an increased risk for IT need on top of dietary and lifestyle measures to control their glycaemia according to the international accepted standards.

These study results should be confirmed, with the measurement of interleukins, serum hCG, FT3 levels, TgAb and other placental factors, to have a better idea of the underlying mechanisms.

## Declaration of interest

K G Poppe is secretary of the European Thyroid Association, received lecture fees from the Berlin-Chemie AG, IBSA and Merck Healthcare KGaA. He is also an Editorial board member for the *European Thyroid Journal*. K G Poppe was not involved in the peer review process of this paper on which he is listed as an author. All authors declare that there is no conflict of interest that could be perceived as prejudicing the impartiality of the work reported.

## Funding

This work did not receive any specific grant from any funding agency in the public, commercial, or not-for-profit sector.

## Author contribution statement

MP and ML draughted the first version of the manuscript. FV collected data and revised the manuscript. PK, GS and LG revised the manuscript. SR revised the manuscript and approved the final version. KGP designed and performed the study, acquired and analysed the data, revised the manuscript and approved the final version.

## Data availability

Data are available upon reasonable request to the corresponding author.
